# A Current Perspective of Two of the Most Aggressive Head and Neck Cancers: Pharyngeal and Laryngeal

**DOI:** 10.3390/curroncol32100572

**Published:** 2025-10-15

**Authors:** Mihaela Iuliana Ciortan (Sîrbu), Maria Alina Marin, Doina Chioran, Iasmina-Alexandra Predescu, Nicolae Constantin Balica, Sergio Liga, Mircea Rivis, Ştefania Dinu, Şerban Talpoş

**Affiliations:** 1 Doctoral School, “Victor Babeș” University of Medicine and Pharmacy Timisoara, 2nd Eftimie Murgu Square, 300041 Timisoara, Romania; mihaela.sirbu@umft.ro; 2 ENT Department, “Victor Babes” University of Medicine and Pharmacy Timisoara, 2nd Eftimie Murgu Square, 300041 Timisoara, Romania; balica@umft.ro; 3 ENT Department, Emergency City Hospital, 400139 Cluj-Napoca, Romania; alina.marin@umft.ro; 4 Faculty of Dental Medicine, “Victor Babeș” University of Medicine and Pharmacy Timisoara, 9 Revolutiei 1989 Ave., 300070 Timisoara, Romania; 5 University Clinic of Toxicology, Drug Industry, Management and Legislation, Faculty of Pharmacy, “Victor Babeș” University of Medicine and Pharmacy Timisoara, 2nd Eftimie Murgu Square, 300041 Timisoara, Romania; sergio.liga96@gmail.com; 6 Research Centre for Pharmaco-Toxicological Evaluation, Faculty of Pharmacy, “Victor Babeș” University of Medicine and Pharmacy Timisoara, 2nd Eftimie Murgu Square, 300041 Timisoara, Romania; 7 Department of Applied Chemistry and Engineering of Organic and Natural Compounds, Faculty of Chemical Engineering, Biotechnologies and Environmental Protection, Politehnica University Timisoara, Vasile Pârvan No. 6, 300223 Timisoara, Romania; 8 Discipline of Oral Surgery, 2nd Department of Dental Medicine, “Victor Babeș” University of Medicine and Pharmacy Timisoara, 2nd Eftimie Murgu Square, 300041 Timisoara, Romania; rivis.mircea@umft.ro; 9 Department of Pediatric Dentistry, Faculty of Dental Medicine, “Victor Babeș” University of Medicine and Pharmacy Timisoara, 9 No., Revolutiei Bv., 300041 Timisoara, Romania; dinu.stefania@umft.ro; 10 Pediatric Dentistry Research Center, Faculty of Dental Medicine, “Victor Babeș” University of Medicine and Pharmacy Timisoara, 9 No., Revolutiei Bv., 300041 Timisoara, Romania; 11 Discipline of Oral and Maxillo-Facial Surgery, Faculty of Dental Medicine, “Victor Babes” University of Medicine and Pharmacy Timisoara, 2nd Eftimie Murgu Square, 300041 Timisoara, Romania; talpos.serban@umft.ro

**Keywords:** head and neck cancer, laryngeal cancer, pharyngeal cancer, risk factors, treatment strategies, early detection

## Abstract

**Simple Summary:**

Pharyngeal and laryngeal cancers are two of the most aggressive forms of head and neck cancer, often diagnosed in advanced stages and associated with high mortality. This review provides an overview of the main risk factors, such as tobacco, alcohol, HPV infection, and dietary habits, as well as typical symptoms and diagnostic challenges. The review also discusses current treatment options, including surgery, radiotherapy, chemotherapy, and immunotherapy. Early detection remains crucial, particularly for cancers that currently lack established screening protocols.

**Abstract:**

Background: Head and neck cancers (HNCs) represent a substantial global health burden, with an estimated mortality rate exceeding 50% annually. Among the various subsites, pharyngeal and laryngeal carcinomas are recognized as two of the most aggressive and challenging forms, characterized by high incidence, poor prognosis, and a strong association with advanced-stage diagnosis. Methods: A systematic literature review was performed using electronic literature databases (e.g., PubMed, Google Scholar). Search terms included “head and neck cancer”, “laryngeal cancer”, and “pharyngeal cancer”. Selected studies are published within the last two decades. Results: Laryngeal cancer constitutes approximately 40% of head and neck malignancies, with a clear male predominance, and pharyngeal cancer shows increased incidence in male populations from the Americas and Africa. Despite therapeutic advancements in radiotherapy, chemotherapy, and immunotherapy, overall survival rates remain unsatisfactory. Moreover, patients are at increased risk for second primary malignancies, particularly within the lungs and esophagus, due to the widespread carcinogenic exposure along the aerodigestive tract. Conclusions: To mitigate the morbidity and mortality associated with pharyngeal and laryngeal cancers, early detection, risk factor mitigation, and public health education are imperative. Enhancing screening among high-risk populations and adopting personalized, multidisciplinary treatment strategies may significantly improve clinical outcomes and long-term survival.

## 1. Introduction

Head and neck cancer (HNC) is a major global health concern, with an estimated 650,000 new cases and deaths reported annually, of which at least 50% are fatal [1]. This malignancy arises from epithelial cells and most commonly affects the upper aerodigestive tract, including the oral cavity, sinonasal region, pharynx, and larynx. In the literature, it is often referred to as head and neck squamous cell carcinoma (HNSCC) [2]. Among these, laryngeal cancer is the most prevalent, accounting for approximately 40% of all head and neck cancers. Each year, around 185,000 new cases are diagnosed, with the highest incidence observed in Spain, Cuba, and Montenegro. Laryngeal cancer primarily affects men over the age of 60, with a 41% death rate compared to women [3]. In contrast, pharyngeal cancer had an incidence of about 3% of all cancer cases in 2001. According to scientific studies, men from African and American regions have been the most affected by oral and pharyngeal cancer, with a mortality rate of approximately 2.6 deaths per 100,000 people between 1994 and 1998 [4].

One of the most common factors that can lead to the development of head and neck cancers over time is the consumption of alcohol and tobacco. Infection with the HPV virus, particularly type 16, accounts for 60% of all reported cases, and its incidence has been continuously increasing. Until the year 2000, the diagnosis of HPV-positive HNC in the United States was steadily growing. However, advances in radiotherapy and chemotherapy have significantly improved survival rates in recent years. Because these carcinogens affect the entire aerodigestive tract, most people diagnosed with head and neck cancer develop secondary tumors in the lungs or esophagus over time [5,6]. Both primary and secondary cancer prevention require early diagnosis and prevention, which are among the most critical population priorities. Implementing them will result in a 50% reduction in the number of cases and deaths reported annually worldwide. Early detection of cases in the population can be achieved through cytology and endoscopy for most types of cancer, including those of the head and neck. Head and neck cancer is often found among smokers, making them especially vulnerable to the risks. However, they choose not to participate in screenings to keep their mental health in good condition [7]. In the United States, by 2025, a total of about 72,680 new cancer diagnoses and 16,680 deaths are estimated for head and neck cancer. For pharyngeal cancer, a total of 59,660 cases is calculated, of which 17,160 are women and 42,500 are men. Estimated deaths from pharyngeal cancer are 12,770, of which 3640 are women and 9130 are men. Meanwhile, for laryngeal cancer, there are an estimated 13,020 new cases, of which 2910 are women and 10,110 are men. Deaths from laryngeal cancer are estimated at 3910, of which 770 are women and 3140 are men. These data are reflected in the information provided by Siegel et al., which shows the incidence and estimated number of deaths associated with these cancers, as shown in the Table 1 below [8]. These statistics underscore the pronounced gender imbalance and high mortality associated with both malignancies.

Following the general overview of head and neck cancers, examining two of the most prevalent forms of malignancy within this region—pharyngeal and laryngeal cancer—is crucial. While both cancers constitute a significant cause of morbidity and mortality globally, each presents distinct clinical challenges and epidemiologic profiles. This article will examine these malignancies, focusing on specific epidemiologic trends, associated risk factors, diagnostic complexity, and therapeutic strategies. This review aims to provide a comprehensive understanding of these cancers and to explore key clinical methodologies to optimize patient management and improve prognostic outcomes.

## 2. Methodology

This article is a narrative review aimed at synthesizing and presenting up-to-date information regarding the diagnosis, risk factors, clinical manifestations, prognosis, and treatment of head and neck cancers, with a specific focus on laryngeal and pharyngeal cancer.

Relevant studies and clinical guidelines were identified through searches in PubMed, Google Scholar, and other significant databases, prioritizing publications from the last two decades. Preference was given to peer-reviewed original research articles, systematic reviews, meta-analyses, and established clinical guidelines, while additional references from specialized books were included when appropriate.

Although this is not a systematic review, the search strategy was conducted in accordance with adapted PRISMA principles, applying predefined eligibility criteria. Inclusion criteria: (i) studies published from 1990 through September 2025, with emphasis on studies published since 2000; (ii) articles addressing laryngeal and/or pharyngeal cancer, particularly squamous cell carcinoma; (iii) studies providing data on epidemiology, risk factors, diagnostic approaches, treatment strategies, prognostic biomarkers, survival outcomes, or quality-of-life measures. Exclusion criteria: (i) conference abstracts or non-peer-reviewed materials lacking complete methodological details; (ii) isolated case reports or minimal case series; (iii) studies focused exclusively on other head and neck cancers without specific details regarding laryngeal or pharyngeal cancer.

This methodological framework ensures a comprehensive yet focused synthesis of the most relevant and high-quality evidence available on the topic.

## 3. Laryngeal Cancer

Laryngeal cancer is a malignant tumor that develops from the epithelial lining of the larynx and is most frequently identified histologically as squamous cell carcinoma (SCC). Globally, it represents around 1–2% of all cancers and accounts for a considerable share of head and neck malignancies, particularly in older men with long-term exposure to tobacco and alcohol. Based on anatomical location, laryngeal tumors are categorized as supraglottic, glottic, or subglottic, with glottic cancers being the most frequent and often associated with earlier symptom onset—most notably persistent hoarseness [9,10,11,12].

The first classification of laryngeal cancers was discovered in 1871 by Sir Morell Mackenzie, who classified them into papillomas, sarcomas, and fibromatosis. Subsequently, in the early 20th century, they were reclassified into carcinomas and sarcomas [13]. Despite advancements in surgical and non-surgical treatment modalities, including radiotherapy and organ-preserving approaches, prognosis is highly dependent on the stage at diagnosis [14]. Early-stage tumors have favorable outcomes, while advanced-stage disease is associated with lower survival rates and a significant impact on speech, airway function, and quality of life. Risk factor modification and early detection remain critical components of effective disease control.

### 3.1. Risk Factors

The development of laryngeal cancer is strongly influenced by a combination of modifiable and non-modifiable risk factors, many of which act synergistically to initiate and promote malignant transformation in the upper aerodigestive tract. For a clearer understanding, risk factors can be classified into two major categories: (i) modifiable (e.g., tobacco, alcohol, occupational exposures, poor oral hygiene, viral infections); (ii) non-modifiable (e.g., age, gender, family history) [9]. This classification facilitates targeted public health interventions and the development of risk-adapted screening strategies.

#### 3.1.1. Tabacco

According to the World Health Organization (WHO), cigarette smoking is responsible for 30% of all cancer deaths, with an estimated over 1,000,000 deaths each year [15]. The larynx is affected by more than 60 toxic substances found in cigarette smoke (e.g., hydrocarbons, aromatic amines, or nitrosamines). When the larynx is exposed to these substances, a cascade of inflammatory reactions is triggered in the human body, which intensifies with prolonged exposure. Over the years, these carcinogens have produced changes in DNA that serve as early indicators of tumors [16]. Joshua Muscat et al. conducted a case–control study on men with primary laryngeal-glottic and supraglottic laryngeal cancer, most of the patients being active smokers. They found that the risk of laryngeal cancer increased with the amount of tobacco consumed. Smokers who smoked more than 20 cigarettes a day had a relative risk 3 times higher for supraglottic cancer and 4 times higher for glottic cancer [17]. The lowest favorable prognosis is observed for supraglottic laryngeal cancer, which spreads very rapidly to lymph nodes and is often diagnosed at a later, more advanced stage [18].

Due to the increased incidence and mortality rate of laryngeal cancer due to smoking, patients must be aware of the risks they expose themselves to daily to prevent the development of more aggressive and harder-to-treat forms of cancer.

#### 3.1.2. Alcohol

Since the early 20th century, alcohol consumption has been recognized as an important public health concern due to its association with laryngeal cancer. In 1987, the International Agency for Research on Cancer (IARC) officially classified alcoholic beverages as carcinogenic, drawing attention to the serious risks that alcohol consumption poses to the human larynx [19]. According to the information available to date, the occurrence of laryngeal cancers is closely related to the proportion of alcohol consumed. In a meta-analysis, Baan et al. found that people who consume 50 g of alcohol daily have a three times higher rate of laryngeal cancer than people who do not consume alcoholic beverages. Most commonly, according to a study of 153 patients, almost 79% of whom were chronic drinkers, alcohol has been shown to contribute to supraglottic and glottic cancers, with the incidence rate of these cancers directly proportional to the amount of alcohol consumed [9,17,20]. When combined with heavy smoking, alcohol consumption dramatically amplifies the risk of developing laryngeal cancer by as much as 177 times compared with individuals who neither smoke nor drink [9]. In a short communication, Bosetti and colleagues analyzed data from two case–control studies investigating the joint effects of alcohol and tobacco use on laryngeal cancer risk. Their analysis included 110 patients with tumors located in the glottis or supraglottis, of whom 40 were non-smokers and 68 did not consume alcohol, and compared them with 160 non-smokers and 161 non-drinkers without cancer. Throughout the hospitalization period, they were monitored and interviewed on their lifestyle and habits concerning smoking and alcohol consumption so that at the end of the analysis, the results showed that non-smoking patients who consumed more than 8 alcoholic drinks per day had a risk almost 3 times higher than those who consumed less alcohol. The risk of laryngeal cancer was also higher among non-alcohol-consuming smokers, with the risk being more significant in those who smoked more than 25 cigarettes a day [21].

#### 3.1.3. HPV

The human papillomavirus, also known as HPV, is a significant risk factor affecting the squamous cells of the skin, nose, vagina, and anus. Out of the hundreds of known subtypes, HPV 16 and 18 are considered the most aggressive forms, causing more than half of HPV cancers worldwide [22]. The mechanism by which HPV invades the body and affects the laryngeal area is complex. HPV activates genes that stimulate cell growth (RAS and epidermal growth factor receptor) and simultaneously blocks genes that suppress cancer cell growth (p53 and Rb), thus promoting tumor development. HPV infection also affects inhibitors of apoptosis and the cell cycle through proteins such as cyclin D1. Measurements of HPV mRNA levels are essential to obtain information about the prognosis of cancer development. For example, the E6 and E7 proteins play a crucial role in cancer development as they activate the cell cycle and inhibit the function of tumor suppressor genes. The progression of laryngeal cancer may be indicated by the increase in expression of markers such as cyclin D1 and E7 proteins, and their analysis can help to diagnose and predict the disease early [23]. Recent meta-analyses indicate that approximately 22–29% of laryngeal cancers are associated with HPV infection, with HPV16 being the most frequently detected subtype [24,25,26].

Immunohistochemical detection of p16 protein is widely used as a surrogate marker of HPV infection in head and neck cancers. p16 functions as a tumor suppressor by inhibiting cell cycle progression, and its overexpression reflects the dysregulation caused by viral oncoproteins E6 and E7. Evidence suggests that p16 status may have prognostic value in laryngeal cancer. In a retrospective study of surgically treated patients, Allegra et al. reported p16 overexpression in 27% of cases. Survival analysis showed markedly better outcomes in the p16-positive group, with 5-year overall survival rates of 90% compared to 29.7% in p16-negative patients, and disease-specific survival of 100% versus 44.4%. These findings indicate that p16 expression not only serves as a marker of HPV-related disease but may also identify patients with a more favorable prognosis [27]. Bueno et al. conducted a systematic literature review on the association between HPV and laryngeal cancer. After evaluating 30 studies that included mainly men with an average age of 60 years, from different geographical regions (e.g., USA, Spain, Italy, China, Mexico, Spain, and Poland), they observed that the HPV-16 subtype was identified as a risk factor in 28 studies and every country, indicating that it is the main factor for HPV infection, while HPV-39 and HPV-56 subtypes had the lowest rate of occurrence in only two studies [28]. In contrast to tobacco consumption, which has the highest occurrence rate of laryngeal cancers and affects people of different ages, HPV infection is most commonly found in young and non-smoking patients [9].

#### 3.1.4. Betel Quid

Betel nuts (BNs) and betel quids (BQs) are widely accepted and used as chewing products. Still, their potentially addictive and psychoactive effects place them in 4th place among the most abused substances worldwide. BN, harvested from the fruit of the Areca catechu palm, can be chewed alone or in combination with tobacco or the fruit of the Piper betel; the combination is commonly referred to as a betel quid. People who consume BQ, both in their free time and at work, do so to achieve pharmacological effects such as feelings of euphoria, increased alertness, a more remarkable ability to concentrate, and improved digestion. Because BQ poses significantly increased risks for oral cancer, the International Agency for Research on Cancer (IARC) includes BQ in class I carcinogens [29,30,31].

#### 3.1.5. Occupational Exposure

Occupational exposure and work in specific industries have also been associated with an increased risk of laryngeal cancer. Substances such as asbestos, sulphuric acid, nickel compounds, diesel and gasoline smoke, wood dust, rubber products, formaldehyde, hair dyes, organic solvents, mineral oils, and coal dust are considered to be the culprit agents. However, the level of scientific evidence varies. Despite strict regulations in some countries, in many parts of Asia, Africa, and Latin America, these substances continue to be widely used, maintaining a high risk of disease [32,33]. According to systematic reviews of published studies, occupational exposure has been associated with an increased risk of laryngeal cancer. Significant associations were observed for exposure to polycyclic aromatic hydrocarbons (PAHs), exhaust fumes, and work in the textile and rubber industries. Workers exposed to PAHs showed a higher risk, especially those in foundries, road construction, or other sectors with frequent use of bituminous substances or carbolineum. In contrast, a moderate but steady increase in risk was reported for exhaust gases. In the textile industry, the risk was greater for factory workers than for simple exposure to textile dust, probably due to contact with additional chemical agents used in the production process. An elevated risk linked to aromatic amines, nitrosamines, or process-related dusts was also noted for employees in the rubber industry. Importantly, the association with PAHs remained significant even after adjusting for smoking and alcohol consumption, and historical exposures, more common before the 1980s, may explain the higher risk observed in older workers who began their occupational activity before this period [34,35]. In a study of non-smoking, non-alcohol-consuming men, the most common occupational exposures reported were to PAHs, diesel exhaust, and solvents. Analyses showed an increased risk for supraglottic tumors associated with exposure to silica and PAHs, as well as further increases in risk for exposure to diesel exhaust and gasoline. An increased risk for glottic tumors was also observed for silica, grain dust, skin dust, and PAH exposure [36]. In a large case–control study using a quantitative job exposure matrix (SYN-JEM), Hall et al. assessed occupational asbestos exposure, respirable crystalline silica, chromium (VI), and chromium (VI) combined with nickel, which were also found to be associated with laryngeal cancer. The highest risks were observed at long durations and high cumulative levels of exposure, particularly in men. For asbestos and silica, the risk was significantly increased at long exposures, and for chromium-VI, the effects were more potent when exposure was isolated, without co-exposure to nickel [37].

Evidence suggests that occupational exposure to various chemical agents and industrial dusts is an independent and important risk factor for laryngeal cancer, with variations in intensity depending on the type of substance and duration of exposure.

### 3.2. Symptomatology

Shephard et al. conducted a case–control study using the UK Clinical Practice Research Datalink (CPRD) database. Patients over 40 years of age, diagnosed with laryngeal cancer between 2000 and 2009, who presented at least once to the specialist in the year before diagnosis were included. The study included 806 cases (678 men and 128 women) and 3559 controls (2960 men and 599 women), with a mean age of 67. Within the 12 months before diagnosis, the most common symptoms reported among laryngeal cancer patients were hoarseness (52%), sore throat (first visit—23%; recurrence—10%), dysphagia (5%), otalgia (4%), recurrent respiratory infections, persistent cough, dyspnea, oral symptoms, and high inflammatory markers. Symptom combinations had a significantly higher predictive value. For example, hoarseness associated with inflammatory markers had a positive predictive value of 15%, while hoarseness and recurrent sore throat reached 12% [38]. According to current National Institute for Health and Care Excellence (NICE) guidelines, the most alarming signs that require emergency medical advice are persistent hoarseness and a throat lump [3]. Furthermore, a retrospective clinical study conducted by Raitiola et al. showed that the presence and severity of symptoms depend on the tumor’s location. In supraglottic cancers, most patients presented with otalgia, dysphagia, and neck pain. Signs that may suggest an advanced form of glottic cancer are otalgia and severe pain on swallowing, with hoarseness being the most common initial symptom caused by vocal cord obstruction [10,39]. In subglottic cancers, the most frequent symptoms are stridor and dyspnea caused by mechanical airway obstruction, often requiring emergency tracheostomy, while hoarseness is less typical [40].

### 3.3. Diagnosis and Prognosis

**Figure 1:**
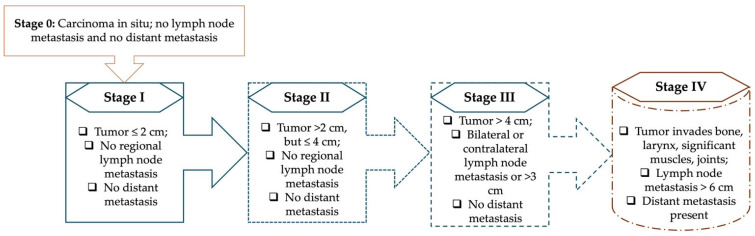
The TNM stages, according to TNM Classification for Head and Neck Cancer [33].

According to the National Comprehensive Cancer Network (NCCN) guidelines, the diagnostic workup for laryngeal cancer should be multidisciplinary and thorough, involving a full otolaryngologic examination, laryngoscopy, swallowing assessment, and imaging studies such as contrast-enhanced computed tomography (CT) and magnetic resonance imaging (MRI) [41]. In clinical practice, accurate staging is a crucial component of patient management and treatment planning. For head and neck malignancies, including laryngeal tumors, the Union for International Cancer Control (UICC) developed the TNM classification system to describe disease extent. Within this framework, “T” denotes the size and invasion of the primary tumor, “N” indicates the status of regional lymph nodes, and “M” reflects the presence of distant metastases [42]. The Figure 1 below shows the TNM stages according to the 8th edition TNM Classification for Head and Neck Cancer [43].

Laryngoscopy represents an essential step in both the diagnosis and staging of laryngeal cancer. Laryngoscopy, with or without local anesthesia, is used to determine the extent of the lesions and the mobility of the vocal cords. Usually, when a detailed examination of tumor spread and deep structures of the larynx, such as the anterior commissure, laryngeal ventricle, and subglottic, is desired, laryngoscopy under general anesthesia is recommended. Also, if the laryngeal cancer is advanced, a detailed endoscopic examination of the esophagus is necessary to rule out the presence of a malignant tumor in the upper portion of the digestive tract, as well as a histopathological examination of biopsies of suspicious lymph nodes. As there is an increased risk of tumor spread to the lymph nodes of the neck, imaging examination is essential. Contrast-enhanced computed tomography (CT) provides a detailed analysis of areas that cannot be visualized by laryngoscopy, such as the subglottis, the spaces surrounding the epiglottis and larynx, and the interior of the thyroid cartilage. After the patient is injected with a biphasic iodinated contrast agent, the examination is performed in a prone position, with the patient breathing normally and in a quiet condition, ensuring clear and precise images. Additional respiration-phase and dynamic maneuvers (e.g., single breath-hold inspiration, puffed cheeks, Valsalva maneuver) may be performed to better evaluate airway dynamics. Multiplanar reconstructions and metal artifact–reducing algorithms are applied to optimize image quality. CT is particularly useful for detecting bone erosion, calcifications, and foraminal enlargement related to perineural spread, and it complements MRI in identifying osseous changes along the skull base foramina [44,45]. A chest CT with contrast or a PET/CT is recommended to check whether the disease has spread to other organs [10,46]; however, due to the high cost, this method is recommended mainly for patients with advanced tumors [41]. Correlation or fusion of PET with MRI and CT significantly improves detection accuracy and delineation of the full extent of perineural tumor spread [44].

The MRI technique, unlike the CT imaging technique, allows for the evaluation of possible soft tissue abnormalities in addition to localizing the cancer, which provides a more detailed delineation of the tumor. This advantage enables laser microsurgery and radiotherapy with enhanced visualization, thereby contributing to more precise interventions with minimal impact on healthy tissue [47]. A study conducted by Murakami et al. analyzed the predictive value of MRI on the long-term local control rate (after 5 years) following radiotherapy, examining 83 patients with early-stage glottic cancer using MR imaging. The analysis results revealed that the 5-year local control rate after radiotherapy was 72%, and the tumor was visible on MRI in 60% of cases. The importance of this information in therapy lies in its ability to provide a more comprehensive assessment of the cancer’s size and its relationship with adjacent structures, which contributes to the selection of the most appropriate treatment [48].

Moreover, MRI provides superior soft-tissue contrast compared to CT and enables better delineation of tumor contours (using T1-weighted sequences without fat suppression), skull base, medullary bone, and laryngeal cartilage infiltration. MRI also helps differentiate tumor tissue from retention sinusitis, assess deep lymph node involvement (retropharyngeal and parapharyngeal nodes), and detect perineural or intracranial tumor spread using T2-weighted, diffusion-weighted, and fat-saturated postcontrast sequences [45]. High-resolution 3T MRI with targeted, contrast-enhanced, fat-suppressed T1-weighted sequences is considered the most appropriate imaging modality for evaluating cranial nerve pathways and perineural tumor extension. According to Lee et al., MRI remains the imaging modality of choice for detecting perineural spread, offering a sensitivity of 95–100% and a specificity of approximately 85%. MRI findings typically include thickening and enhancement of the involved cranial nerves, obliteration of perineural fat planes, and widening of skull base foramina. These characteristics, when correlated with CT or PET/CT, provide a comprehensive overview of both the anatomic and metabolic extent of disease, which is essential for accurate staging and treatment planning [44].

To determine the prognosis for laryngeal cancers, it is essential to consider patient factors, including (i) age, (ii) gender, (iii) functional status, as well as (iv) the location and extent of the tumor. When laryngeal cancer is diagnosed in its earlier stages and the tumor is small and accessible, the prognosis is good, with a cure rate up to 95%. Intermediate lesions have a variable prognosis, depending on the tumor’s location, size, extent of invasion into nearby tissues, and its spread to the surrounding lymph nodes [49].

### 3.4. Treatment

#### 3.4.1. Current Guideline-Based Treatments

Current treatment guidelines for laryngeal cancer aim to preserve the larynx [50]. In the early stages (I–II), conservative surgical approaches, such as transoral laser microsurgery and partial laryngectomy, or radiotherapy, provide excellent oncological control while maintaining function. In advanced stages (III–IV), total laryngectomy with adjuvant radiotherapy or chemoradiotherapy remains standard, although organ-preservation strategies based on concurrent CRT may be considered in selected patients. Surgical therapy is also indicated in cases of recurrence after radiotherapy or chemoradiotherapy. For patients unfit for surgery, targeted therapies or palliative chemotherapy can be used [51]. Radiotherapy is a globally accepted and recommended treatment method for localized cancers and can provide symptomatic relief in advanced stages. As radiotherapy is based on radiation, strict dose control is necessary to minimize the risk of toxicity, with a maximal deviation of 5% being tolerated [52]. Building on these findings, Adeel et al. evaluated long-term outcomes in a retrospective analysis of 242 patients with stage I and II laryngeal cancer treated with radiotherapy. Of these, 92% were men, 8% were women, and 57% were smokers. All patients were re-evaluated approximately 5 years after definitive radiotherapy, with a statistically higher survival rate for stage I (87%) compared to stage II (67%). Moreover, local disease control at 5 years was 84% for stage I and 71% for stage II. Treatment duration did not have a significant impact on the survival rate. Overall, after undergoing radical radiotherapy treatments, more than 50% of patients were estimated to be saved [53].

The American Society of Clinical Oncology guideline update by Forastiere et al., based on a systematic review of 150 studies published between 2005 and 2017, provided updated recommendations on larynx-preservation strategies in the treatment of laryngeal cancer. Following the results obtained, they proposed evidence-based guidelines for the safe and effective use of endoscopic surgical resection in patients with early-stage laryngeal cancer while recommending total laryngectomy only in patients with metastatic disease. They suggested using positron emission tomography to examine vocal function and swallowing post-treatment [54].

Given the variability in treatment strategies across different professional societies, a comparison of guideline-based recommendations is essential. Table 2 provides an overview of the main approaches proposed by the NCCN, ESMO, and ASCO for the management of laryngeal cancer according to disease stage.

As shown in Table 2, all guidelines converge on radiotherapy or conservative surgery for early-stage disease, while concurrent chemoradiotherapy and total laryngectomy remain the standard for advanced stages. Minor differences exist regarding the emphasis on organ-preservation strategies and the role of induction chemotherapy. Overall, guideline consensus highlights the balance between oncological safety and functional outcomes. This comparison reveals broad agreement on treatment for both early and advanced stages but also identifies some differences in organ-preservation strategies.

#### 3.4.2. Organ-Preservation and Conservative Surgical Strategies

Considering the role of radiotherapy in the treatment of early-stage laryngeal cancer and its impact on survival rate, an alternative approach that has gained attention is the laryngeal preservation method, which has also been investigated as suitable for patients with early-stage laryngeal cancer. This treatment is performed by open partial laryngectomy or transoral laser microsurgery, when the tumor can be removed, leaving the free margins with healthy tissue intact [50]. In severe cases of advanced laryngeal cancer, when the tumor invades adjacent tissues and organs, the treatment option is total laryngectomy (TL). This involves the complete removal of the larynx, often with neck dissection and, in some cases, thyroidectomy. Permanent tracheostomy and loss of natural voice are inevitable consequences of TL, while the most frequent postoperative complications include wound infection and pharyngocutaneous fistula. The risk of fistula is particularly high in previously irradiated patients, where vascularized tissue flaps are selectively recommended to lower this risk. In the more advanced stages, the most effective therapeutic option may be a combination of chemotherapy and radiotherapy, as this approach has been shown to preserve pharyngeal integrity and laryngeal function, making it a viable alternative when surgical treatment is not feasible for medical reasons [50,52].

Non-surgical treatments may include KTP laser and photodynamic therapy (PDT). The potassium titanyl phosphate (KTP) laser is a treatment option with a success rate of up to 96% in early glottic tumors, where no nearby tissues have been invaded. It destroys the tumor’s vasculature using 532 nm light, which is absorbed by oxyhemoglobin. Photodynamic therapy (PDT) is a treatment option for non-advanced glottic cancer, combining a photosensitizing agent with cold laser light to destroy tumor cells, with a proven efficacy of up to 82% [57]. Another minimally invasive CO2 laser technique for tumor resection is Transoral Laser Microsurgery (TLM). This endoscopic method enables the precise cutting of tumor tissue without affecting surrounding healthy areas. It is indicated in laryngeal tumors in early stages (T1, T2, T3), providing good results over time. Although very well tolerated, TLM is not entirely free of postoperative complications. Patients may experience bleeding, respiratory obstruction, dental sensitivity, burns of the oral cavity, reversible neuropathy, tongue edema, or, in severe cases, tongue paresis [58]. To assess whether transoral laser microsurgery (TLM) with the potassium-titanyl-phosphate (KTP) laser (TLM-KTP) offers an additional benefit in treating early glottic cancer, Parker et al. analyzed 88 patients with metachronous glottic malignancies. In the first phase, they quantified the survival rate, observing that no patient experienced complications following treatment, resulting in a 100% survival rate in this treatment group. Vocal function was also monitored, yielding excellent results and underscoring the well-established benefits of TLM-KTP [59].

Open partial laryngectomies (OPHLs) play an essential role in the conservative treatment of laryngeal cancer. They provide the link between transoral approaches and total laryngectomy or other organ-preserving methods, instrumental in cases of recurrent and radio-resistant laryngeal carcinoma. Traditionally, OPHLs were divided into three groups: vertical (for glottic carcinomas, with resection of the vocal cords and anterior commissure), horizontal (for supraglottic), and atypical (such as subtotal laryngectomy or combined open and endoscopic techniques). However, the European Laryngological Society (ELS) has proposed a modern classification based on the cranio-caudal direction of resection, and it is presented in Table 3 [60].

#### 3.4.3. Systemic and Emerging Therapies

Despite having the standard of care, traditional treatments such as methotrexate, bleomycin, cisplatin, and 5-fluorouracil are only effective temporarily. They can only reduce the tumor by up to 30% when administered in individual doses. Thus, for patients with metastases in regions distant from the initial cancer site, the best treatment option is the combination of Platinum and 5-fluorouracil with Cetuximab, which has shown the highest increase in survival rate. Alternatively, Cisplatin with Paclitaxel or 5-fluorouracil can be safely used [61]. An experimental preclinical study by Sim MW and his collaborators evaluated the antitumor potential and toxicological profile of cisplatin administered in vitro and in vivo in combination with HA polysaccharide, a ligand for cancer cell-specific marker receptors. Based on studies showing that this injectable combination has a high potential to reduce toxicity compared to standard cisplatin-based therapy, these researchers injected in vivo cancer cells into the larynx of mice to create a laryngeal cancer xenograft and then administered HA-cisplatin. The research results demonstrated a superior anti-tumor profile of the HA-cisplatin combination in vivo compared to standard cisplatin therapy, with one of the mice being completely cured by the end of the experiment, showing no residual tumor [62].

Furthermore, molecular medicine is gaining increasing relevance in everyday clinical practice as sequencing technology becomes widely available to guide personalized treatment choices in laryngeal cancer and prevent overly aggressive therapies, indicating, if necessary, the need to adjust treatment intensity. Some studies have determined the prediction of response to targeted treatments by integrating genetic markers, such as the p53 protein, which is involved in regulating the cell cycle and protecting cell DNA, and the EGFR (Epidermal Growth Factor Receptor) protein, which regulates cell growth [57,63,64,65]. Other important biomarkers in evaluating metastases include β2-microglobulin, HIF-1α, and HIF-2, which play a significant role in assessing disease progression, along with NLR, PTEN, and cyclin D1. Drugs inhibiting PD-L1 protein, a protein capable of suppressing immune responses, include pembrolizumab and nivolumab. These treatment options help the body recognize and combat cancer cells, thereby improving the survival rates of patients with head and neck cancer [66].

## 4. Pharyngeal Cancer

Pharyngeal cancer encompasses a group of malignant tumors arising from the mucosal lining of the nasopharynx, oropharynx, and hypopharynx, each with distinct epidemiological, etiological, and clinical features. Most of these cancers are squamous cell carcinomas, reflecting their origin in the stratified squamous epithelium. Although pharyngeal cancer is less common than other head and neck malignancies, it is considered one of the most aggressive, often diagnosed at advanced stages due to its deep anatomical location and non-specific early symptoms [67,68]. Given the complex and multifactorial nature of pharyngeal carcinogenesis, understanding the risk profile of affected individuals is essential for both prevention and early detection. The following section provides a structured overview of the modifiable risk factors associated with pharyngeal cancer. To illustrate these differences more clearly, Table 4 summarizes the main risk factors, clinical features, diagnostic approaches, and treatment strategies across pharyngeal subsites (NPC, OPSCC, HPC).

### 4.1. Nasopharyngeal Carcinoma (NPC)

#### 4.1.1. Risk Factors

##### Epstein–Barr Virus (EBV)

Approximately 96% of nasopharyngeal carcinoma (NPC) cases in endemic regions are attributed to the Epstein–Barr virus (EBV), which causes lifelong asymptomatic infection in most of the population. In NPC, EBV exhibits type II latency, with viral proteins and microRNAs disrupting cellular pathways, inducing genetic instability, and evading immune surveillance, which favors tumor progression. The risk of NPC is influenced by age at primary EBV infection, with infection in childhood potentially leading to incomplete immune control and cancer development in adulthood. Variability in EBV strains, including deletions or amino acid substitutions in viral genes (e.g., LMP1, BALF2, BNRF1, RPMS1), has been correlated with oncogenesis and may represent a target for therapeutic interventions [70].

##### Dietary

In endemic regions, dietary habits play a significant role in carcinogenesis. The most incriminated foods are salted fish, preserved meats, salted vegetables, and smoked foods, all significantly associated with increased NPC risk. Among these, salted fish has shown the strongest correlation. In contrast, a regular intake of fresh dark-green vegetables exerts a protective effect. Thus, the traditional diet, rich in salted and nitrosamine-preserved foods, is considered a key etiological factor in NPC, while the consumption of fresh vegetables may contribute to its prevention [81].

#### 4.1.2. Symptomatology

The symptoms of nasopharyngeal carcinoma (NPC) vary depending on the stage and tumor extension, the most common being nasal symptoms (unilateral obstruction, epistaxis, nasal voice, smell disturbance), present in about 80% of patients. Hypoacusis, recurrent otitis media, tinnitus, and sensation of auricular fullness occur at the otic level. At the same time, tumor extension may cause neurological manifestations by affecting cranial nerves (vestibulo-cochlear, facial, and abducens). Cervical or submandibular adenopathies are frequently associated, and headache, low-grade fever, anemia, and weight loss may occur. In the early stages, signs are non-specific and of low intensity, but as the tumor progresses, symptoms worsen and may indicate intracranial invasion. NPC also has an increased tendency for distant metastasis, most often to bone, liver, lung, and lymph nodes [73]. According to a retrospective study, in patients with nasopharyngeal carcinoma, the first symptoms are usually in the ear (hearing loss, otitis, sensation of a blocked ear), followed by swelling in the throat and nasal signs such as nasal obstruction or bleeding. It can sometimes be mistaken for sinusitis, an allergy, or a throat infection. More rarely, the disease manifests as headaches or neurological disturbances such as double vision or numbness in the face. The diversity of symptoms means that diagnosis is often delayed [82].

#### 4.1.3. Diagnosis and Prognosis

NPC is often diagnosed at advanced stages due to its deep anatomical location and non-specific symptoms, making early detection and accurate prognostication essential for improving patient outcomes. In recent years, significant progress has been made in both diagnosis strategies and prognosis assessment, combining imaging, serology, molecular markers, and advanced analytic tools. MRI has gradually replaced CT as the preferred modality for staging of the primary tumor and regional nodes, given its superior soft tissue resolution. At the same time, PET-CT remains valuable for detecting distant metastases [83]. MRI has also shown higher sensitivity than endoscopy in detecting lesions, including those invisible on endoscopic examination, highlighting its potential role in early screening protocols [76]. On the molecular side, plasma Epstein–Barr virus (EBV) DNA detected by quantitative PCR has demonstrated remarkable sensitivity (93–97%) and specificity (98%), outperforming serological IgA antibody tests [84]. To further improve early detection, studies have explored epigenetic biomarkers: Chiang et al. reviewed circulating cell-free DNA methylation panels, such as RASSF1A and DAPK1, which enhanced the detection of early-stage NPC when combined with EBV DNA [85]. Hsu et al. validated RERG methylation in plasma cfDNA as a promising biomarker, with a sensitivity of 80% and a specificity of 100% [86]. In addition, Ye et al. reported that combining plasma HSP90α with EBV VCA-IgA and EBV DNA significantly improved diagnostic accuracy, achieving an AUC of 0.954 compared to individual markers [84]. Complementing these approaches, Fijardo et al. highlighted that miRNA signatures, including EBV-encoded miRNAs, hold promise as future diagnostic and prognostic tools [87].

Beyond diagnosis, several biomarkers and clinical parameters have been identified as strong prognosticators in NPC. Chiang et al. systematically reviewed over one hundred studies and concluded that plasma EBV DNA, tumor and nodal volumes, and systemic inflammatory markers such as the neutrophil-to-lymphocyte ratio (NLR) and CRP/albumin ratio consistently correlated with survival outcomes [85]. Also, it is further demonstrated that elevated plasma HSP90α and EBV DNA before treatment predicted worse survival, whereas a post-treatment decline was associated with improved prognosis [84]. In pediatric populations, Shi et al. found that TNM stage and radiotherapy were independent prognostic factors, with a five-year overall survival rate of 89.3% in patients receiving radiotherapy compared to 68.6% in those without [88]. Importantly, Jiang et al. emphasized that early detection translates into excellent long-term outcomes, with five-year disease-specific survival rates exceeding 90% in patients diagnosed at early stages [83].

#### 4.1.4. Treatment

The standard therapy for non-proliferative carcinoma (NPC) and a crucial intervention for non-disseminated NPC is radiotherapy. Radiation therapy (RT) alone is recommended for stage I cases in the early stages, and RT in conjunction with chemotherapy is recommended for stage II patients. It is advised in these situations to use radiation therapy directed at the primary tumor and a few chosen cervical lymph nodes. For locally advanced disease with lymph node metastases, CRT is also the standard treatment. The combination of radiation and chemotherapy demonstrated significantly improved treatment control in patients with T3-4N0-1 [79].

### 4.2. Oropharyngeal Squamous Cell Carcinoma (OPSCC)

#### 4.2.1. Risk Factors

##### HPV

HPV infection represents the main risk factor for oropharyngeal squamous cell carcinoma (OPSCC), particularly HPV-16. HPV-positive patients are typically younger, with less exposure to tobacco and alcohol, higher socioeconomic status, and a predominance of males. Oral HPV is considered a sexually transmitted infection, with an increased risk observed in individuals reporting a high number of lifetime sexual partners. The oropharynx, especially the tonsils and base of tongue, appears particularly susceptible due to the presence of transitional mucosa that facilitates viral persistence. Importantly, epidemiological studies have demonstrated that HPV infection confers a distinct biological profile compared to HPV-negative OPSCC, underscoring its etiological role [74].

##### Dietary

Beyond alcohol-related risks, dietary patterns have been shown to play a critical role in the etiology of oral and oropharyngeal cancers. Several studies have reported that high consumption of red meat and eggs may be associated with an increased cancer risk, whereas dairy products, poultry, fish, and citrus fruits such as tangerines appear to exert a protective effect. Among nutritional factors, the most significant risk reduction—estimated at up to 50%—has been observed in individuals with a diet rich in fruits and vegetables, a benefit likely attributable to their antioxidant and anti-inflammatory components. Regular consumption of vitamins C and E has also been associated with a lower incidence of cancer. Conversely, vitamin D supplementation has shown a paradoxical link to increased risk, although additional studies are required to understand this relationship better [89,90].

#### 4.2.2. Symptomatology

Uzcudun et al. conducted a retrospective study and identified the most frequently observed specific symptoms of pharyngeal cancer as dysphagia, malnutrition, odynophagia, otalgia, neck mass, neck lymph nodes, constitutional symptoms, and voice changes [91]. Similarly, a study by Kamstra et al. described additional symptoms associated with pharyngeal cancer, including xerostomia, decreased sensitivity in the mouth, tongue, and other areas of the oral cavity, decreased mouth motility, and an inability to wear dentures [66]. Due to the non-characteristic symptoms of cancer, pharyngeal cancer is often misdiagnosed as pharyngitis or tonsillitis [92]. A study conducted between 2008 and 2013 investigated the initial presenting symptoms in patients diagnosed with oropharyngeal squamous cell carcinoma. The most frequently reported manifestations were cervical mass and neck pain, present in approximately 77% of cases. Additional symptoms included dysphagia, globus sensation, odynophagia, and otalgia. Notably, the distribution of symptoms differed based on HPV tumor status. Patients with HPV-positive tumors were more likely to present with a painless cervical mass, whereas HPV-negative patients more frequently experienced pain-related symptoms, such as neck pain, odynophagia, dysphagia, bleeding, and unintentional weight loss. These findings suggest that HPV status not only impacts prognosis and treatment response but also influences the clinical presentation, which may have implications for early detection and differential diagnosis [93].

#### 4.2.3. Diagnostic and Prognosis

In the management of treatment and patient care for pharyngeal cancer, accurate staging is equally essential as for laryngeal cancer.

The Union for International Cancer Control’s (UICC) TNM staging classification is also applicable to evaluate pharyngeal cancer, which plays a crucial role in determining tumor spread and planning appropriate treatment.

In the first phase of diagnosing pharyngeal cancer, the doctor will evaluate the oral cavity using the mirror examination technique. An intraoral examination is more precise than palpation due to the early stages of lesions being small but profound, and it provides more information about the mucosa [77]. A definitive diagnosis of pharyngeal cancer requires a biopsy. Histopathological evaluation using conventional light microscopy with hematoxylin–eosin staining remains the standard approach, allowing classification of carcinoma into three grades: (i) well-differentiated (G1), (ii) moderately differentiated (G2), and (iii) poorly differentiated (G3). Although immunohistochemistry and electron microscopy are not routinely employed, they may help characterize undifferentiated tumors [46].

Accurate staging is essential in OPSCC, as in all head and neck cancers. The AJCC 8th edition recognizes the distinct clinical and prognostic implications of HPV status in OPSCC and therefore introduced a separate staging system for HPV-positive tumors. Thus, HPV-associated OPSCC benefits from a different staging and therapeutic approach compared to HPV-negative OPSCC, the differences being explained by the biological characteristics of the cancer as well as the specific patient profile [94]. The main differences between HPV-positive and HPV-negative OPSCC are summarized in Table 5 [94].

To support these clinical and prognostic differences, modern imaging and molecular methods play an essential role in diagnosis and staging. PET/CT is used to stage and subsequently follow up patients with oropharyngeal cancers. Although its sensitivity for micrometastases is low, PET/CT is particularly recommended for patients with locally advanced disease or for identifying the primary lesion in unknown primaries. In treatment monitoring, PET/CT is useful for detecting early recurrence or residual disease and for adapting therapeutic strategies accordingly [95]. Recent research has introduced several methods to verify HPV involvement in OPSCC tissue, including PCR, RT-PCR, p16 immunohistochemistry, and in situ hybridization using DNA or RNA. These diagnostic tools allow classification of OPSCC as either HPV-positive (HPV+) or HPV-negative (HPV−). Current clinical guidelines recommend evaluating HPV status in all OPSCC cases, using validated assays to detect active infection and identify high-risk HPV types in head and neck cancers [96].

In diagnosing HPV-dependent cancers, ultrasonography plays a significant role in identifying lymph node metastases based on abnormal lymph node features, especially in tumors with an unknown primary site. Node size is an indirect indicator of malignancy, with size values of 10 mm for cervical nodes in the axial plane. The shape of malignant lymph nodes is usually round, and their margins are sharper than in normal nodes [97].

#### 4.2.4. Treatment

In the early stages, when the tumor is small and has not invaded nearby tissues and organs, the treatment options for patients with oropharyngeal cancer are radiotherapy or surgery. In contrast, combining both methods is used in more advanced stages of analysis [98]. Due to the highly invasive nature of surgery, which involves the removal of healthy tissue to reach the tumor, many treatment options have shifted towards non-surgical approaches. These procedures aim to view the tumor through the mouth without harming the mandible and surrounding tissues, using laser microsurgery (TLM), laryngoscopic video-operated surgery (LVS), robotic surgery (TORS), endoscopic laryngopharyngeal surgery (ELPS), and ultrasound surgery (US). All these new treatment methods have demonstrated a significant decrease in hospitalization time and the risk of post-surgical complications and have been incorporated into the current NCCN treatment guidelines [80].

IMRT, also known as Intensity-Modulated Radiation Therapy, is a radiotherapy technique that uses a three-dimensional system to target tumors and deliver high doses without affecting healthy tissue. It is ideal for the treatment of head and neck cancers, including pharyngeal cancers, because all organs adjacent to tumors are sensitive, so IMRT provides much less radiation exposure to them [99]. In addition to its minimally invasive effects, IMRT is particularly well-suited for preserving the function of the salivary glands, which are susceptible to injury during radiotherapy. Once damaged, this leads to xerostomia, or dry mouth, which affects taste, swallowing, and speech processes. All studies investigating the benefits and impact of IMRT compared with conventional radiotherapy have shown that IMRT contributes significantly to improving patients’ quality of life [100]. A 6-year study supported the benefits of IMRT treatment for patients with squamous cell carcinoma of the oropharynx, as well as the importance of chemotherapy treatment. The LRC treatment control achieved a score of 92% among the 107 patients tested. However, distant metastases remained the predominant pattern of failure, underscoring the importance of effective systemic therapy to improve overall survival, especially considering that 80% of patients received concurrent platinum-based chemotherapy [101]. Systemic palliative chemotherapy is the next step in treating pharyngeal cancer if locoregional therapies like surgery and radiotherapy have not been effective in improving the patient’s condition. The chemotherapy method can involve a single drug, such as methotrexate, cisplatin, bleomycin, or 5-fluorouracil, or a combination of them. However, the most effective treatment combination is the administration of a dose of 800–1000 mg/m^2^/day for 96–120 h of 5-Fluorouracil with 80–100 mg/m^2^ cisplatin bolus on day 1, which has demonstrated a response rate of 70% [102]. In a trial conducted over 6 years on patients suffering from oropharyngeal cancer, it was demonstrated that neoadjuvant chemotherapy with cisplatin was superior compared to only local-regional treatment and no chemotherapy. Treatment with cisplatin (100 mg/m^2^ on day 1) followed by a continuous 24 h intravenous infusion of 5-FU (1000 mg/m^2^/day) improved median survival to 5.1 years, compared with 3.3 years in the control group. Although event-free survival was reported as zero in both cohorts, the neoadjuvant chemotherapy group demonstrated borderline significance (*p* = 0.11) [103].

In addition to advances in radiotherapy, another therapeutic approach is immunotherapy, also known as adoptive T-cell therapy, which targets the patient’s T-cells to combat the cancer. These cells are obtained from blood or tumor tissue, cultivated, and modified in the laboratory to enhance their effectiveness in combating cancer. This type of treatment is effective in HPV cases, as the proteins produced by this virus are suitable therapeutic targets for immunotherapy [104].

The therapeutic approach to oropharyngeal cancer has evolved significantly over time, combining strategies such as IMRT, chemotherapy, and immunotherapy to improve patient survival and quality of life. The choice of optimal treatment depends on disease stage and individual patient characteristics, and new personalized therapies offer promising prospects for increasing efficacy and reducing toxicity.

### 4.3. Hypopharyngeal Carcinoma (HPC)

#### 4.3.1. Risk Factors

##### Genetic Predisposition

Familial cancer is one way of understanding the causes of cancer, indicating the possibility of inherited genes being involved. It is essential to counsel patients on familial genetics before initiating treatments to assess the risk of developing cancer and predisposing factors [105]. Goldstein and co-workers investigated whether a family history of cancer affects the risk of developing oral and pharyngeal malignancies, analyzing epidemiological data from a case–control study. Their findings indicated that individuals with a family history of cancer had an increased likelihood of pharyngeal cancer, especially when family members had a history of cancer, mainly caused by smoking. Risks were higher when cancer occurred in siblings, especially males, compared to females [106]. Furthermore, the International Head and Neck Cancer Epidemiology (INHANCE) has analyzed how family history, alcohol, and tobacco may influence the risk of head and neck cancers in a population of people aged under and over 45 years. They found that younger patients have a higher risk of developing head and neck cancers due to alcohol and smoking, with genetic factors playing a significant role in older patients’ development [107].

##### Alcohol

Alcohol is among the key risk factors for the development of oral and pharyngeal cancers, accounting for roughly 30% of cases [108]. Over the past several decades, numerous studies have explored this association, consistently showing a clear dose–response effect. The risk of pharyngeal cancer rises significantly with increasing alcohol intake, with daily consumption of 25, 50, or 100 g of pure alcohol linked to progressively higher risks. Individuals who drink more than five alcoholic beverages per day—especially when combined with smoking—face up to a fivefold greater likelihood of developing pharyngeal carcinoma compared with non-drinkers. Furthermore, the anatomical site of the tumor appears to correlate with the regions most exposed to direct contact with alcohol, with the hypopharynx being especially vulnerable to alcohol-induced epithelial damage [109]. Koyama et al. investigated if alcohol consumption in patients with pharyngeal cancer could contribute to an increase in mortality over time. They gathered 2626 patients from a cancer hospital database in Japan, followed them for 10 years, and determined their alcohol consumption levels by dividing them into non-users, past users, and occasional, moderate, or chronic drinkers. According to the results obtained, the poorest prognosis of survival was for men and women chronic consumers of alcohol (>46 g ethanol/day), with an estimated life expectancy of 1114 days, while at the opposite pole, for non-consumers and occasional consumers, it was 2800 and 2101 days, respectively [110]. In a meta-analysis, Kabat et al. demonstrated that alcohol, regardless of its source (e.g., wine, beer), in combination with smoking, significantly contributes to the development of pharyngeal cancer. In women, drinking more than four drinks per day and smoking more than 20 cigarettes was found to increase the risk 27-fold compared to women who did not smoke and did not consume alcohol. In men, those who consumed more than seven drinks and smoked more than 31 cigarettes daily had a 20-fold higher risk [111].

#### 4.3.2. Symptomatology

The most frequent presenting symptom of hypopharyngeal cancer is dysphagia, reported in nearly half of patients. Other common manifestations include odynophagia, dysphonia, and the sensation of a pharyngeal foreign body. Because the hypopharynx has a highly developed lymphatic network, tumors in this region tend to spread rapidly to cervicojugular and retropharyngeal lymph nodes, which increases the likelihood of distant metastasis. As a result, approximately 6% of patients are diagnosed with metastatic disease at presentation, and up to 60% develop metastases during or after treatment [75,112].

#### 4.3.3. Diagnostic and Prognosis

HPC is one of the rarest but most aggressive head and neck cancers, often diagnosed at an advanced stage due to its hidden location and nonspecific early symptoms. Recent research has focused on improving both diagnostic accuracy and prognostic stratification to guide treatment and preserve laryngeal function. Kim et al. compared larynx-preserving surgery (LPS) with upfront radiation therapy in early-stage HPC and reported superior 5-year disease-free survival and functional outcomes after LPS, especially for cT2 tumors [78]. Fan et al. study demonstrated that 18F-FDG PET/MR outperformed conventional CT and MRI in T and N staging, achieving 90.9% and 71.4% accuracy, respectively, and further showed that diffusion parameters such as ADCmean were predictive for both survival and laryngeal preservation [113]. Zhang et al.’s study applied single-cell and bulk transcriptomics to characterize the tumor microenvironment and identified gene modules and immune cell compositions that could predict treatment resistance, offering potential biomarkers for prognosis and therapy stratification [114].

Several studies have investigated survival determinants and treatment effects. Burbure et al., analyzing over 9000 patients from the NCDB (National Cancer Database), found that survival for stage I/II disease was not influenced by treatment modality, but surgery conferred a survival advantage in T4 tumors. They also showed that HPV-positive tumors, present in ~21% of cases, were associated with significantly better overall survival (HR 0.60) [115]. Répássy et al. similarly reported that HPV/p16 expression did not significantly affect survival in their Hungarian cohort, but patients treated with partial pharyngolaryngectomy achieved the most extended median survival of over 75 months [116]. Pala et al. evaluated long-term outcomes of patients treated with (chemo)radiotherapy and found a 5-year OS of only 24%. Their multivariate model identified surgery before RT, chemotherapy, comorbidity score, pretreatment tracheostomy, hemoglobin levels, and initial treatment response as independent prognostic factors [117].

Taken together, these findings suggest that multimodal imaging, especially PET/MR, and molecular profiling of the tumor microenvironment may enhance early diagnostic precision and prognostic assessment. Prognosis is determined not only by stage and HPV status but also by host-related factors such as comorbidity and systemic inflammatory markers. While chemoradiotherapy remains the backbone of organ-preserving treatment, selected patients benefit from surgery, particularly in advanced T-stage disease.

#### 4.3.4. Treatment

For hypopharyngeal cancer (HPC), the main therapeutic objective is to preserve laryngeal function without compromising oncologic control. Over time, this has led to the refinement of innovative surgical techniques and the development of carefully tailored chemoradiotherapy and radiotherapy regimens [28]. According to the most recent ESMO Pan-Asian guidelines, treatment varies by stage of disease. For early-stage disease (T1–T2N0M0), definitive radiotherapy or conservative surgery with laryngeal preservation is recommended, with RT or CRT administered if required. In T1–T2N0M0 and T3N0–3M0 tumors, the standard of care is concurrent CRT targeting both the primary tumor and nodal disease [IV, A]. If surgery, including laryngectomy, becomes necessary, it is usually performed after CRT, or following induction chemotherapy in cases of partial or complete response; if the disease remains stable or progresses, surgery may also be indicated. For T4A N0–M0 cancers, radical surgery without preservation of the larynx remains the treatment of choice, generally followed by RT or CRT [IV, A]. However, other strategies may include CRT [IV, B], radiotherapy in responders to induction therapy, or salvage surgery if the disease fails to regress [I, A]. Finally, in T4B N0–3 disease, options are more limited and include induction chemotherapy followed by RT in responders, concurrent CRT [IV, B], or palliative strategies, such as systemic chemotherapy, immunotherapy, palliative radiotherapy, or best supportive care [118,119,120].

## 5. Prevention and Public Health Strategies

The global incidence of head and neck squamous cell carcinoma has been rising significantly every year worldwide, specifically among young individuals, with an estimated increase in cases of around 30% by 2030 [121].

Scientific research has shown that lifestyle and screening play a crucial role in preventing cancer and reducing related mortality rates. Studies have demonstrated that adopting a healthy lifestyle can reduce the risk of cancer of any type by up to 50%, highlighting the negative effects of smoking and obesity, which are the most prevalent risk factors. Head and neck cancers can be prevented by HPV vaccination, increasing screening, reducing alcohol and tobacco consumption, and adopting a healthy diet and maintaining good oral hygiene. Additionally, in secondary prevention, particularly for oral cancers, dentists in the US are legally required to perform oral examinations on patients seeking medical services, enabling the earlier diagnosis of potential oral cancers. Thus, if patients were made aware of the risks of an unhealthy lifestyle, cancer mortality could fall dramatically. Therefore, if patients managed to become much more responsible about their lifestyle and recognize the risks they face daily, cancer mortality would decrease significantly [121,122].

Smoking is the predominant risk factor for oral and pharyngeal cancers, accounting for over 75% of cases in Western Europe. Current evidence indicates that smokers face up to a 25-fold greater likelihood of developing these malignancies compared with non-smokers. Importantly, research has shown that the risk decreases substantially after smoking cessation, even among long-term smokers [123]. A review by Khalifeh et al., which analyzed studies published up to 2022, further supports this association. The authors compiled 65 studies on smoking and head and neck cancers—most originating from Italy, the USA, Spain, and France—with 35 included in a meta-analysis assessing dose–response effects. Their findings revealed that former smokers have a 0.4-fold higher relative risk of oral, pharyngeal, and laryngeal cancers compared with current smokers. In the dose–response meta-analysis, the relative risk was 0.47 for every 10 years of smoking cessation, decreasing significantly with increasing age. This study demonstrated that smoking cessation significantly decreases the risk of head and neck cancers, even within the first 5 years of abstinence, particularly in Europe and North America, emphasizing the importance of smoking cessation in preventing the development of cancers [124]. Later, in 2023, Gaikwad and his collaborators aimed to identify smoking cessation strategies and their effects on people with head and neck cancers in randomized controlled trials. Searching popular databases such as PubMed, Google Scholar, and EBSCO and applying inclusion and exclusion criteria, they analyzed five papers published, mostly in the United States, between 2016 and 2021. According to the results, one of the studies showed that married people are most likely to quit smoking. In contrast, another study showed that 30% of patients who received a recommendation to read a special book were more likely to cease smoking. While only two studies reported significant increases in cessation rates following interventions, further studies are needed to identify the most appropriate ways for patients to quit smoking easily and more effectively [125]. Similarly, Caini et al. further explored this topic in a systematic literature review based on the results of 16 studies, including 13 studies comparing the survival rate of patients who were smokers and ex-smokers and three studies comparing the hazard ratio (HR) of survival of each group with a reference group (e.g., former long-term smokers or non-smokers). Most included studies originated from North America, and the majority of patients were between 55 and 70 years of age. They were diagnosed with glottic, laryngeal, or oropharyngeal cancers at all stages (from early to advanced) and were followed for 2 to 27 years. The results showed that the survival rate increased by 20% with smoking cessation before or in the immediate period close to diagnosis, as well as a longer survival with very reliable recurrence control [126].

The most studied smoking cessation method is nicotine substitution in cigarettes [127]. This therapy is recommended for patients who want to quit smoking, providing optimal and safe control of a gradual reduction in addiction to cigarettes. The process of quitting smoking is a long-term one, and once patients suddenly stop, they may experience withdrawal symptoms and very intense cravings, which will make the process difficult. For this reason, nicotine replacement therapy has become one of the main methods of prevention in the development of head and neck cancer [128]. Chewing gum was the first type of nicotine substitute on the market. It is available in doses of 2 mg and 4 mg, while in some countries, the lowest strength is available over-the-counter medication. Its use can be limited, however, by oral and gastric side effects, reduced absorption in combination with coffee or acidic drinks, incorrect dosing, and the risk of addiction in certain situations. Therefore, to mitigate the effects associated with nicotine chewing gum, alternative forms of substitutes have been developed, including transdermal patches, nicotine lozenges, sprays, and inhalers. Transdermal patches can deliver nicotine in doses ranging from 5 to 52.6 mg over 16 or 24 h. Nicotine patches are available in doses of 1 mg, 1.5 mg, 2 mg, and 4 mg. Nasal sprays and inhalers are available in doses of 0.5 mg or 1 mg per puff, respectively, as well as 10 mg and 15 mg per dose. In all these substitutes, the amount of nicotine absorbed is significantly lower than the doses of nicotine released from cigarettes, making them suitable for gradually reducing nicotine dependence and facilitating smoking cessation [129,130]. Regarding medical interventions to help patients with head and neck cancer quit smoking, Lena and Tishelman discussed the effectiveness of nicotine substitution. Before starting therapy, patients were given trial products to replace cigarettes, which included nicotine patches, chewing gum, lozenges, or Swedish snuff of varied concentrations. For 10 weeks, patients received the chosen product and were evaluated throughout the entire administration period. One of the patients noted that the nicotine lozenges helped him suppress his cravings, while another said the patches helped to relieve the dizziness caused by nicotine withdrawal. Initially, many patients were doubtful about the effectiveness of nicotine replacement therapy (NRT) in helping them quit smoking. Over time, however, its use has become widespread, with evidence showing that NRT significantly increases cessation rates [127]. In this context, Rennard et al. conducted a double-blind, randomized trial involving 420 smokers motivated to quit, assessing the impact of a nicotine inhaler on smoking reduction. During the 15-week treatment, some of the patients received nicotine inhalers (Nicorette 10 mg) in doses of 6–12 cartridges per day, and some received inhalers without nicotine (placebo). At the end of the treatment, the nicotine inhaler-treated group had a significant reduction in smoking compared with the placebo group, with 18% of participants in the active group reducing smoking by at least 50% compared with 8% in the placebo group at 4 months. The mean reduction in smoking was 74% for subjects who reduced by 50% in the active group. Smoking cessation rates were higher in the nicotine inhaler group, and significant improvements were seen in smoking-related symptoms and quality of life. Symptoms that showed the greatest improvement were coughing, phlegm, the senses of smell and taste, and breathing. The results of the study suggest that nicotine inhalers can be safely used to reduce smoking, with the smokers included in the study being able to significantly reduce their tobacco consumption while also demonstrating the health benefits of nicotine inhalation itself [131]. Nicotine transdermal nicotine patches have also been evaluated over time to demonstrate their effectiveness in smoking cessation. Fiore and colleagues carried out a meta-analysis of studies evaluating nicotine patches to determine their overall effectiveness and optimal application in treating tobacco dependence. Seventeen studies were reviewed, comparing 16 h versus 24 h release patches, treatment duration, discontinuation strategies (abrupt cessation vs. tapering), and success rates at the end of therapy and six months afterward. The findings demonstrated that nicotine patches were significantly more effective than placebo, with abstinence rates of 27% at treatment completion and 22% at six months, compared with 13% and 9% in the placebo group. The 24 h patch was associated with higher abstinence rates compared to the 16 h patch, and treatments lasting more than 8 weeks showed greater long-term efficacy. No significant differences were observed between abrupt and gradual discontinuation of treatment; however, gradual dose tapering had a positive impact on maintaining abstinence at 6 months. More frequent, intensive behavioral counseling significantly improved success, but the nicotine patch was also effective with minimal counseling. These results suggest that, in general, the nicotine patch is an effective option for helping smokers quit, even without intensive behavioral counseling. However, more intensive counseling may significantly improve success rates, but it is not essential to achieve substantial benefits [132].

Nicotine’s harmful effects extend to almost every organ in the body, and it is the leading cause of 20% of reported annual deaths in the US, costing over $300 billion. Although there are medications and substitution therapies that help people quit smoking, all healthcare professionals must strongly encourage patients to quit, even if only a small percentage succeed [133].

HPV is implicated in the development of genital, anogenital, and head and neck cancers, with a particularly strong association with oropharyngeal cancer, whose incidence is rising worldwide. In the United States, nearly 70% of oropharyngeal cancer cases are HPV-positive, most often affecting younger Caucasian men [134]. The growing burden of HPV-related oropharyngeal cancers (HNSCCs) has been linked to shifts in sexual behavior, with studies from both the USA and Europe indicating earlier sexual debut and a higher number of sexual partners. For example, the percentage of women who had sex before the age of 18 increased from <10% in 1900–1920 to 60% in the 1970s. In the same decades, risky sexual behaviors increased, leading to greater exposure to oral HPV. 86% of men aged 30–40 years reported having oral sex, compared with 74% of those aged 50–69 years and 62% of those aged 70 years or older, and among women, 82%, 77%, and 43% of the same age groups reported the same behavior, respectively [135]. As of January 2020, more than 104 countries had implemented HPV vaccination, with higher coverage in high-income countries compared to low-income countries. Vaccination programs conducted in schools achieved higher coverage than those conducted in health facilities. In the United States, coverage initially resembled that of other adolescent vaccines but increased more slowly compared to them. The high cost of the vaccine also limited its introduction in many countries. Still, the Global Alliance for Vaccines and Immunization (GAVI) has supported vaccination in low- and middle-income countries [136].

The table below (Table 6) provides a comparative overview of the three FDA-approved HPV vaccines (e.g., Cervarix, Gardasil, Gardasil 9) highlighting their targeted HPV genotypes, manufacturers, approved clinical indications, recommended vaccination schedules, and the timeline of regulatory approvals. This information is particularly relevant in the context of HPV-associated malignancies, including oropharyngeal cancers, for which vaccination may offer significant preventive benefits.

Chaturvedi et al. investigated the prevalence of oral HPV in young U.S. adults (ages 18–33), comparing vaccinated and unvaccinated individuals using data from the National Health and Nutrition Examination Survey (NHANES) collected between 2011 and 2014. The study aimed to determine the effect of HPV vaccination on oral infections with high-risk types linked to oropharyngeal cancer. Mouthwash samples were tested by PCR and genotyped for HPV types 16, 18, 6, and 11. Findings revealed a significantly lower prevalence of oral HPV infections among vaccinated participants compared with those unvaccinated (0.11% vs. 1.61%, *p* = 0.008), corresponding to an estimated 88.2% reduction. The protective effect was especially notable in men (0.0% vs. 2.13%, *p* = 0.007). Overall, vaccination was estimated to have prevented roughly 169,650 infections with these HPV subtypes, accounting for 17.0% of potentially preventable infections at the population level, with a stronger effect in women (25.0%) than in men (6.9%) [137].

Similarly, Herrero and colleagues assessed the efficacy of the bivalent HPV vaccine against oral HPV-16 and HPV-18 infection in a randomized trial involving 7466 women aged 18–25 years. Participants received either the bivalent vaccine or a control vaccine in three doses (at baseline, one month, and six months). At the four-year follow-up, 5840 women (91.9% of eligible participants) provided oral samples. The overall prevalence of oral HPV was low (1.7%), yet the difference between groups was marked: 15 HPV-16/18 infections were detected in the control group versus only one in the vaccinated group, yielding an estimated vaccine efficacy of 93.3%. In comparison, the efficacy against cervical infection with HPV-16/18 in the same cohort was 72.0% (*p* = 0.04). These results demonstrate that vaccination provides strong protection against oral HPV infection, highlighting its role as one of the most effective preventive strategies for HPV-associated oropharyngeal cancer [138]. Taken together, these studies confirm that HPV vaccination substantially reduces oral HPV infection and represents a key intervention in preventing HPV-driven oropharyngeal cancer. Since many cases are detected at locally advanced stages, screening functions as a secondary prevention strategy that lowers mortality rates without altering disease incidence. Given the long progression of cancer, screening enables the detection of premalignant lesions at an early stage, allowing for the early identification of these lesions, slowing disease progression, and facilitating earlier curative treatment. Cytology-based approaches, such as those used in cervical cancer screening, have not shown effectiveness in head and neck cancer. In contrast, repeated visual oral examinations demonstrated long-term benefits in reducing both incidence and mortality from oral cancer in a randomized trial conducted in India. These findings support the adoption of oral visual screening, particularly in high-incidence regions and among individuals with tobacco and alcohol use. In addition, serologic detection of HPV antibodies (anti-HPV 16) has emerged as a valuable biomarker for identifying HPV-related oropharyngeal cancer. In studies to date, individuals who subsequently developed oropharyngeal cancer were found to have detectable antibodies up to 10 years before diagnosis. However, there are still many issues that need to be addressed to enable the early detection of potentially malignant lesions in HPV-positive patients [139,140].

## 6. Conclusions

Laryngeal and pharyngeal cancers remain a major global oncologic burden due to their aggressive nature, late-stage diagnosis, and significant functional impact. Despite therapeutic advances, survival rates are still largely dependent on the timeliness of diagnosis and access to multidisciplinary care. Tobacco use, excessive alcohol consumption, and HPV infection continue to be the most influential modifiable risk factors. As such, public education, HPV vaccination, and behavioral prevention strategies play a pivotal role in reducing disease incidence.

Early detection remains the cornerstone of improved prognosis, particularly since early-stage tumors respond favorably to treatment. However, advanced-stage disease often requires complex multimodal approaches, which may carry substantial functional and psychosocial consequences. Rehabilitation—including speech, swallowing, and psychological support—should be considered integral to comprehensive cancer care. Ongoing progress in molecular diagnostics, liquid biopsy, and personalized medicine is paving the way toward more precise and effective interventions. Population-based screening approaches may contribute to identifying optimal prevention strategies; however, their feasibility and cost-effectiveness still require further evaluation. To improve patient outcomes, continued research, expanded screening strategies, and a focus on prevention and long-term quality of life are essential.

Although progress has been made, several uncertainties remain. At present, there are no validated population-based screening programs for laryngeal or pharyngeal cancers, and their feasibility still needs to be assessed. The prognostic value of HPV in sites other than the oropharynx, such as the larynx or hypopharynx, is not yet clearly established and requires larger prospective studies. Similarly, while organ-preservation approaches have improved quality of life, questions remain regarding patient selection and long-term oncologic safety. In addition, promising tools such as liquid biopsy and immune-related biomarkers need further validation before they can be translated into routine clinical use.

Ultimately, this review aims to serve as a clinically relevant synthesis for healthcare professionals and practitioners, providing a practical reference for the prevention, diagnosis, treatment, and long-term management of patients affected by laryngeal and pharyngeal cancers.

## Figures and Tables

**Table 1:** Estimated incidence and mortality of pharyngeal and laryngeal cancers by gender.

Type of Cancer	Estimated Total (Cases)	Women	Men	Estimated Total (Deaths)	Women	Men	Reference
Pharyngeal Cancer	59,660	17,160	42,500	12,770	3640	9130	[8]
Laryngeal Cancer	13,020	2910	10,110	3910	770	3140	[8]

**Table 2:** Comparison of Guideline-Based Recommendations for Laryngeal Cancer Management.

Stage/Strategy	NCCN	ESMO	ASCO	Reference
**Early stage (I–II)**	RT or conservative surgery (TLM, partial laryngectomy)	RT or transoral surgery	Endoscopic resection or RT; preserve larynx	[54,55,56]
**Locally advanced (III–IV)**	Concurrent CRT (organ preservation); TL + adjuvant RT/CRT	Same as NCCN; emphasize CRT	CRT recommended; TL reserved for advanced cases	
**Recurrent disease**	Salvage surgery if feasible	Salvage surgery if feasible	Salvage surgery if feasible	
**Metastatic/unresectable**	Systemic therapy (platinum + cetuximab, immunotherapy)	Systemic therapy	Clinical trials, immunotherapy	

**Table 3:** Classification of Open Partial Laryngectomies (OPHLs) According to the European Laryngological Society (ELS).

OPHL Type	Main Resection	Variants/Extensions	Reconstruction Method
**Type I Supraglottic**	Removal of the entire supraglottis (pre-epiglottic space + upper half of thyroid cartilage) down to the anterior commissure and ventricular folds.	+ARY → includes one arytenoid +BOT → includes base of tongue +PIR → includes part of the piriform sinus	Thyro-hyoidopexy (or thyro–tongue base pexy if hyoid bone is resected)
**Type II Supracricoid**	Removal of the entire thyroid cartilage, inferior limit = upper cricoid ring.	IIa → with crico-hyoido-epiglottopexy (CHEP), sparing suprahyoid epiglottis IIb → with crico-hyoidopexy (CHP), removing the entire epiglottis +ARY → extended to one arytenoid (applies to IIa or IIb)	CHEP (IIa) or CHP (IIb)
**Type III Supratracheal**	Removal of supraglottic, glottic, and part of the subglottic larynx, sparing at least one functioning crico-arytenoid unit. Inferior limit = cricoid plate and/or first 1–2 tracheal rings.	IIIa → with tracheo-hyoido-epiglottopexy (THEP) IIIb → with tracheo-hyoidopexy (THP) +CAU → extended to one crico-arytenoid unit	THEP (IIIa) or THP (IIIb)

**Table 4:** Comparative overview of clinical characteristics, risk factors, and management strategies for nasopharyngeal (NPC), oropharyngeal (OPSCC), and hypopharyngeal carcinoma (HPC).

Aspect	Nasopharyngeal Carcinoma (NPC)	Oropharyngeal Squamous Cell Carcinoma (OPSCC)	Hypopharyngeal Carcinoma (HPC)	References
**Major risk factors**	Epstein–Barr virus (EBV) infection; salted/preserved foods (fish, meat, vegetables); genetic susceptibility	Human papillomavirus (HPV, mainly HPV16); tobacco and alcohol; sexual behavior	Tobacco and alcohol (synergistic effect); poor nutrition; genetic predisposition	[69,70,71,72]
**Typical symptoms**	Nasal obstruction, epistaxis, hyponasal speech; hearing loss, otitis media, tinnitus; cervical lymphadenopathy; cranial nerve palsies in advanced stages	Painless cervical mass (HPV+); dysphagia, odynophagia, otalgia, voice changes; weight loss (HPV−)	Dysphagia, odynophagia, referred otalgia; neck mass; late diagnosis due to nonspecific symptoms	[73,74,75]
**Diagnosis & prognosis**	EBV DNA testing; nasoendoscopy with biopsy; MRI/CT for local extension; high rate of distant metastases	HPV testing (p16 IHC, PCR, ISH); biopsy; PET/CT for staging; prognosis better for HPV+ tumors	Laryngoscopy and biopsy; imaging for staging; often diagnosed at an advanced stage; poor prognosis	[76,77,78]
**Treatment**	Radiotherapy ± chemotherapy (platinum-based); surgery less common	Surgery (TORS, TLM) or radiotherapy for early stages; chemoradiotherapy for advanced disease; immunotherapy in HPV+ cases	Surgery (often extensive, e.g., laryngopharyngectomy) with neck dissection; adjuvant RT/CRT; IMRT to preserve function; palliative chemo in advanced cases	[28,79,80]

**Table 5:** Key distinctions between HPV-positive and HPV-negative OPSCC according to the AJCC 8th edition.

Characteristic	HPV-Positive OPSCC	HPV-Negative OPSCC
Typical patient profile	Younger age at diagnosis, often <60 years; higher proportion of males; strong association with sexual behavior	Older patients, usually >60 years; more frequently male; strong history of tobacco and alcohol exposure
Ethnicity	More common in white populations	More evenly distributed, but still higher in some high-risk groups
Risk factors	HPV16 is the most frequent subtype, linked to multiple sexual partners and oral HPV exposure	Tobacco and alcohol are predominant causal factors.
Tumor characteristics	Frequently arises in the tonsils and base of tongue; often, early nodal involvement even in small primaries.	It can occur at any oropharyngeal site; it is usually diagnosed at an advanced local stage.
Histology	Non-keratinizing, basaloid morphology	Conventional keratinizing SCC
Staging (AJCC 8th ed.)	Classified separately due to distinct biology and prognosis, stage grouping is generally more favorable.	Staged as other HNSCCs; no downstaging benefit.
Prognosis	Better overall survival and disease-specific survival; HPV positivity is a favorable prognostic factor	Poorer survival; higher disease-specific mortality
Molecular profile	Alterations in DNA damage response genes, PI3K pathway, and immune-related gene expression	Frequent TP53 mutations; disruption of cell-cycle regulators (e.g., CDKN2A, RB1); oxidative stress pathways often altered

**Table 6:** Summary of FDA-Approved Human Papillomavirus (HPV) Vaccines: targeted HPV types, indications, vaccination schedules, and regulatory approval history.

Vaccine	HPV Types Targeted	Manufacturer	Indications	Vaccination Regimen	FDA Approval Timeline	References
Cervarix	HPV 16, 18	GlaxoSmithKline	Females aged 9–25 years for the prevention of cervical cancer Cervical intraepithelial neoplasia grade 1, 2 Adenocarcinoma in situ	3 doses: 0, 1, 6 months	2009: females aged 9–25 years old	[134,136]
Gardasil	HPV 6, 11, 16, 18	Merck & Co	Females aged 9–26 years old for prevention of cervical, vulvar, vaginal, and anal cancer, genital warts, and precancerous or dysplastic lesions	3 doses: 0, 2, 6 months	2006: females aged 9–26 years old, 2009: males aged 9–26 years old	[134,136]
Gardasil 9	HPV 6, 11, 16, 18, 31, 33, 45, 52, 58	Merck & Co	Females aged 9–45 years old for prevention of genital warts, precancerous or dysplastic lesions, cervical, vulvar, vaginal, anal, oropharyngeal, and other head and neck cancers	2-dose series: 0, 6–12 months (for ages 9–14), 3-dose series: 0, 2, 6 months (for ages 15–45)	2014: females aged 9–26 years old and males aged 9–15 years old 2015: expanded for males aged 16–26 years old 2018: expanded for individuals aged 27–45 years old 2020: for prevention of specific HPV-related head and neck cancers	[134,136]

## Data Availability

The original contributions presented in the study are included; further inquiries can be directed to the corresponding author.
